# Controlling hypersonic boundary layer transition with localized cooling and metasurface treatments

**DOI:** 10.1038/s41598-024-66867-4

**Published:** 2024-07-10

**Authors:** Furkan Oz, Kursat Kara

**Affiliations:** School of Mechanical and Aerospace Engineering, Oklahoma State University, Stillwater, OK 74078 USA

**Keywords:** Aerospace engineering, Mechanical engineering

## Abstract

This study investigates a novel method to control hypersonic boundary layer transition using a combined local cooling and local metasurface treatment. The method’s effectiveness was investigated on a 5-degree half-angle blunt wedge with a nose radius of 0.0254 mm at a freestream Mach number of 6.0 using direct numerical simulations and linear stability theory. We explored four cases: (i) adiabatic baseline case, (ii) locally cooled case, (iii) local metasurface case, and (iv) combined local cooling-local metasurface case. Results showed that the combined local cooling-local metasurface treatment significantly reduced both wall pressure disturbance amplitude and the density perturbation amplitude around the sonic line, indicating a potential for controlling hypersonic boundary layer transition. In the local cooling-local metasurface case, the disturbance amplitude at the end of the computational domain was 270 times lower than the baseline case. The study also examined the impact of Reynolds numbers, ranging from 25.59 million per meter to 32.80 million per meter. Unsteady simulations revealed that the Reynolds number had a negligible effect on the local cooling-local metasurface performance, indicating that the proposed method applies to a wide range of flight conditions.

## Introduction

The transition of the hypersonic boundary layer from laminar to turbulent states is crucial for designing efficient hypersonic vehicles. This transition impacts vehicle performance in several significant ways. Firstly, aerodynamic heating increases substantially during this transition, which modern thermal protection systems can mitigate, but at a higher cost and often reduced payload capacity, as well as necessitating frequent maintenance^1–3^. Additionally, the transition affects viscous drag, which in turn negatively impacts the vehicle’s overall aerodynamic performance^4,5^. Beyond these thermal and aerodynamic effects, the boundary layer transition can also influence the overall operability of the aircraft. Consequently, there is significant interest in developing a boundary layer transition control system capable of stabilizing this transition.

Understanding the growth of instability waves is crucial for stabilizing the boundary layer, as various disturbance sources are present in the flow environment. Depending on their physical characteristics, there are two different naming conventions widely accepted in the transition community. One convention uses Mack’s first and second modes^6^, while another classifies waves as fast (F) or slow (S) modes based on phase speed behavior near the leading edge^7^. Both naming conventions are discussed in the literature^8,9^.

In hypersonic boundary layers, instabilities associated with Mack’s first and second modes are critical for the transition to turbulence. Existing hypersonic vehicles often rely on thermal protection systems tailored to short-term flights. However, for sustained hypersonic flight, the drawbacks of these systems necessitate finding better alternatives. Therefore, extensive research focuses on understanding and controlling Mack’s modes to delay the transition and reduce the need for heavy thermal protection systems. One proposed method is wall cooling, which stabilizes the first mode but destabilizes the second and higher modes at hypersonic speeds^6,10–18^. Since the second mode is the dominant instability in hypersonic boundary layers for slender geometries, cooling the entire wall is ineffective. Local wall cooling or heating has shown potential in mitigating the destabilizing effects of full wall cooling^17,19–28^.

Research on local wall cooling demonstrates that it stabilizes the first mode without amplifying the second mode. However, due to the second mode’s dominance at hypersonic speeds, Malmuth et al.^29^ proposed an ultrasonically absorptive coating (UAC) to stabilize it. This porous surface has been extensively investigated^28–43^. Studies confirm that porous surfaces stabilize the second mode significantly while only slightly destabilizing the first mode^39^. The existing literature uses porous surface or ultrasonically absorptive coating terms to refer to the surfaces that have simple pore openings. On the other hand, the term metasurface, which covers more complex micro- or nano-scale openings, is a more general definition for such surfaces. Hence, in this study, porous surfaces with various shapes (e.g., circular, rectangular, hexagon-shaped pores) are referred to as metasurfaces^38,44–48^.

Our previous studies investigated the effects of local cooling boundary conditions, cooling strip length, and location on boundary layer transition on an axisymmetric cone^17^. We later examined the stabilization of metasurfaces using semi-transparent wall boundary condition implementation and compared the results with the resolved metasurface region with the immersed boundary method^49^ over the axisymmetric cone. Due to the strong stabilization obtained in the previous studies, we first proposed a local cooling-local metasurface (LC-LM) boundary layer transition control method and presented our preliminary results for 2D wedge geometries^28^. Later, we investigated LC-LM stabilization over the axisymmetric geometries and showed the importance of the metasurface region end effects^50^. This manuscript extends our previous work^28^ by combining local cooling with a local metasurface region to study the stabilization mechanism over a wedge, presenting new analyses and comprehensive results on boundary layer transition control. Detailed discussions on flow physics and the correlation between Reynolds number and stabilization characteristics are included, providing deeper insights and highlighting novel contributions beyond our initial study.

**Figure 1:**
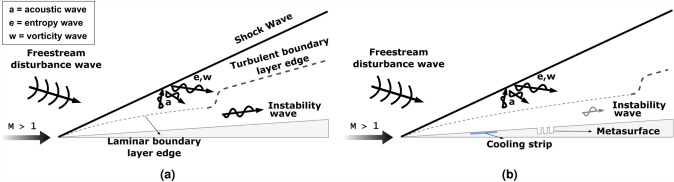
Schematic illustration of complex flow physics and disturbance growth: (**a**) Over a solid wall, leading to an early transition to turbulent flow and increased heat load, and (**b**) using a local cooling and metasurface treatment, delaying boundary layer transition and reducing heat load.

The schematic description of the study is illustrated in Fig. 1. We apply the local cooling-local metasurface (LC-LM) treatment to a 5-degree half-angle wedge with a nose bluntness of 0.0254 mm (0.001 inches) at a freestream Mach number of $M=6$ using direct numerical simulations (DNS). Our in-house high-fidelity solver, OK-DNS^17,28,51^, is employed to obtain flow field variables. OK-DNS is a compressible Navier–Stokes solver that incorporates a fifth-order accurate weighted essentially non-oscillatory (WENO) scheme^52^ for spatial discretization and a third-order total variation diminishing (TVD) Runge–Kutta scheme^53^ for time integration. We compare DNS results with those obtained from our in-house linear stability solver, OK-LST^17,28,51^, which employs a second-order finite difference method for initial guess generation and a fourth-order compact difference scheme for the eigenvalue spectrum.

For the first time, we examine the effect of Reynolds number on the LC-LM boundary layer transition control method. Our numerical simulations revealed that the LC-LM method could reduce the amplitude of instability waves by up to 270 times compared to a scenario without boundary layer transition control, potentially leading to a significant delay in transition. Moreover, changes in the Reynolds number did not severely affect the performance of the LC-LM method.

## Governing equations

This study solves the Navier–Stokes equation in the following form:

1 $$\begin{aligned} \frac{\partial Q}{\partial t}+\frac{\partial A}{\partial x}+\frac{\partial B}{\partial y}=\frac{\partial A_{v}}{\partial x}+\frac{\partial B_{v}}{\partial y}, \end{aligned}$$

where *Q* is the state vector, *A* and *B* are *x*- and *y*-direction inviscid flux vectors given by:

2 $$\begin{aligned} Q= \begin{bmatrix} \rho \\ \rho u \\ \rho v \\ \rho E \end{bmatrix}, \hspace{6pt} A= \begin{bmatrix} \rho u \\ \rho u^{2}+p \\ \rho uv \\ (\rho E+p)u \end{bmatrix}, \hspace{6pt} B= \begin{bmatrix} \rho v \\ \rho vu \\ \rho v^{2}+p \\ (\rho E+p)v \end{bmatrix}, \end{aligned}$$

and $A_{v}$ and $B_{v}$ are *x*- and *y*-direction viscous and heat conduction flux vectors, which are calculated by:

3 $$\begin{aligned} A_{v}= \begin{bmatrix} 0 \\ \tau _{xx} \\ \tau _{xy} \\ u \tau _{xx} + v \tau _{xy} - q_{x} \end{bmatrix}, \hspace{6pt} B_{v}= \begin{bmatrix} 0 \\ \tau _{yx} \\ \tau _{yy} \\ u \tau _{yx} + v \tau _{yy} - q_{y} \end{bmatrix}. \end{aligned}$$

The viscous stresses and heat fluxes have the following form:

4 $$\begin{aligned} \tau _{xx}= & {} \frac{2}{3} \frac{\mu }{Re} \begin{pmatrix} 2\frac{\partial u}{\partial x}-\frac{\partial v}{\partial y} \end{pmatrix}, \end{aligned}$$

5 $$\begin{aligned} \tau _{yy}= & {} \frac{2}{3} \frac{\mu }{Re} \begin{pmatrix} 2\frac{\partial v}{\partial y} - \frac{\partial u}{\partial x} \end{pmatrix}, \end{aligned}$$

6 $$\begin{aligned} \tau _{xy}= & {} \tau _{yx} = \frac{\mu }{Re} \begin{pmatrix} \frac{\partial u}{\partial y}+\frac{\partial v}{\partial x} \end{pmatrix}, \end{aligned}$$

7 $$\begin{aligned} q_{x}= & {} -\frac{\gamma }{(\gamma -1)PrRe} \frac{\partial T}{\partial x}, \end{aligned}$$

8 $$\begin{aligned} q_{y}= & {} -\frac{\gamma }{(\gamma -1)PrRe} \frac{\partial T}{\partial y}. \end{aligned}$$

Herein, (*x*, *y*) represents the two-dimensional coordinates, (*u*, *v*) are the corresponding velocity components, $\rho$ is density, and *p* is pressure. The total energy, *E*, is, therefore:

9 $$\begin{aligned} E=c_{v}T+\frac{u^{2}+v^{2}}{2}, \hspace{12pt} p=\rho RT, \end{aligned}$$

where *T* is the temperature. The viscosity, $(\mu )$, is computed using Sutherland’s law, and the coefficient of conductivity is given in terms of the Prandtl number $(Pr = 0.7)$. For computational purposes, the equations are transformed from the physical coordinate system (*x*, *y*) to a computational curvilinear coordinate system $(\xi , \eta )$. The details of the transformation and transformed governing equations are given in Ref.^17^. The resulting system of equations is solved using OK-DNS, which employs a fifth-order accurate weighted essentially non-oscillatory scheme in spatial discretization and a third-order accurate total variation diminishing Runge–Kutta method in time discretization.

The direct numerical simulations consist of two steps. In the first step, the steady mean flow is computed using variable time stepping until the residuals reach machine zero. Following this, the unsteady computations are performed by superimposing acoustic perturbations to the mean flow at the outer boundary, and the resulting disturbance fields are calculated. The acoustic perturbations at the outer boundary are expressed as:

10 $$\begin{aligned} p^{\prime }={\text {Real}}\left\{ \tilde{p} e^{i \alpha x \pm i \beta y-i \omega t}\right\} . \end{aligned}$$

Here, $\alpha$ and $\beta$ represent the wave numbers in the *x* and *y* directions, respectively, and $\omega$ is the frequency of the acoustic disturbance. The wave number in the *y*-direction is assumed to be zero, corresponding to a zero-degree incident angle. For a detailed understanding of disturbance generation and implementation in the DNS code, interested readers can refer to the references^17,54–56^. This study uses linear stability code to cross-validate the results obtained with the OK-DNS. For the linear stability code, physical quantities are discretized as follows:

11 $$\begin{aligned} \phi (t,x,y) = {\bar{\phi }}(t,x,y)+{\tilde{\phi }}(t,x,y), \end{aligned}$$

where $\phi (t,x,y)$ corresponds to flow parameters, such as *u*, *v*, *p*, $\rho$. Substituting these equations into the governing equations yields the linearized perturbation equations. After some simplifications, we may represent velocity, pressure, and temperature in harmonic waveform as:

12 $$\begin{aligned} {\tilde{\phi }} = {\hat{\phi }}(y)e^{i(\alpha x+\beta z- \omega t)}. \end{aligned}$$

**Figure 2:**
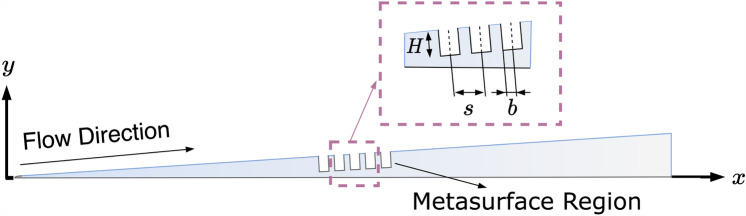
Schematic visualization of metasurface dimensions on a wedge in hypersonic flow.

Herein, hat notation, such as ${\hat{\phi }}$, corresponds to the amplitude of the instability wave. After the simplification and substitutions to the Navier–Stokes equations, the resultant system of equations is solved with OK-LST with relevant boundary conditions. For implementation details and extensive validations, readers are referred to References^17,28,49,50^. Both OK-LST and OK-DNS codes use a semi-transparent boundary condition implementation for the metasurface region (see Fig. 2 for example metasurface shape). The metasurface boundary condition is implemented replacing the wall-normal velocity boundary condition with the pore admittance as follows:

13 $$\begin{aligned} v_{w, n}=p_w^{\prime } {\text {Real}}\left( A_m\right) -\frac{1}{\omega } \frac{\partial p_w^{\prime }}{\partial t} {\text {Imag}}\left( A_m\right) , \end{aligned}$$

where $v_{w, n}$ is the wall-normal velocity, $p_w^{\prime }$ is the wall pressure disturbance, and $A_m$ is the admittance. The wall pressure disturbance is calculated as $p_w^{\prime }=p_w-p_{w,s}$, where $p_{w, s}$ is the wall pressure calculated in the steady flow.

## Results

**Figure 3:**
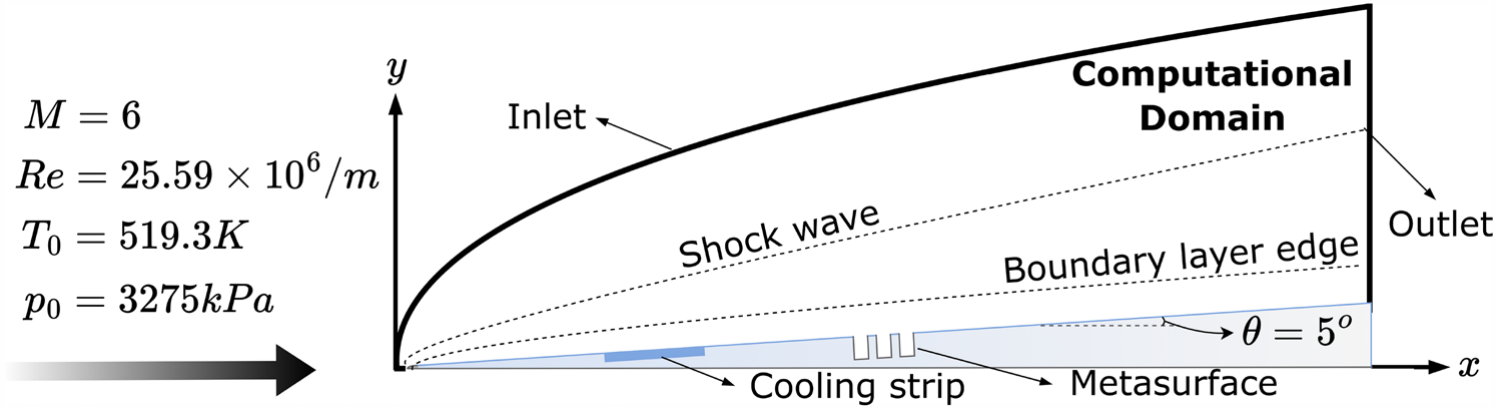
Sketch of the computational domain with flow and boundary conditions.

The hypersonic boundary layer will be investigated using local cooling (LC), local metasurface (LM), and local cooling-local metasurface (LC-LM) treatment. To investigate the stability, our OK-DNS code^12,17,57^ will be run for a flow over a 2D 5-deg half-angle wedge with small nose bluntness, where the nose tip radius is 0.0254 mm (0.001 inch). The freestream Mach number is 6, and the freestream temperature is 63.3 K. The corresponding unit Reynolds number is $25.59 \times 10^6/$m. The schematic description of the flow with LC-LM treatment is illustrated in Fig. 3. The ratio of the adiabatic wall temperature to the freestream temperature was 7.052. The flow conditions and the wall temperature are based on the experimental study of Horvath et al.^58^. The employed wall-to-freestream temperature corresponds to the adiabatic wall temperature in Horvath et al.’s experiment. Thus, the simulation with this ratio will be called the adiabatic wall case hereafter.

**Figure 4:**
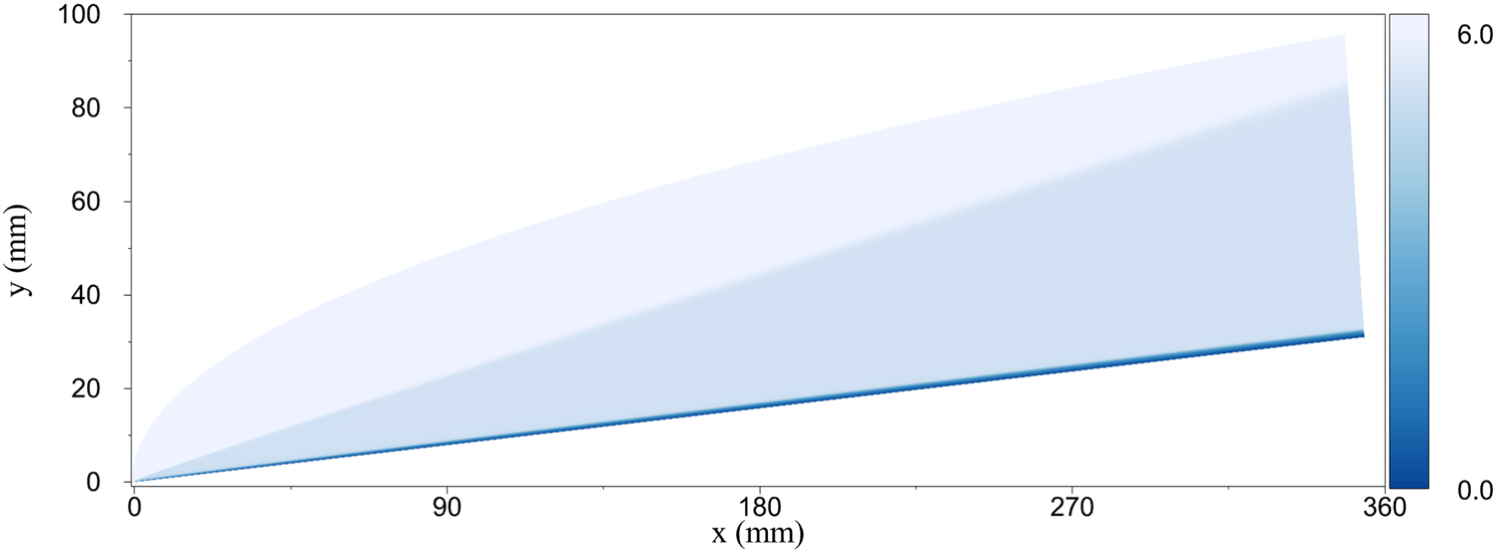
The Mach number contour showing the computational domain for the adiabatic wall case.

**Figure 5:**
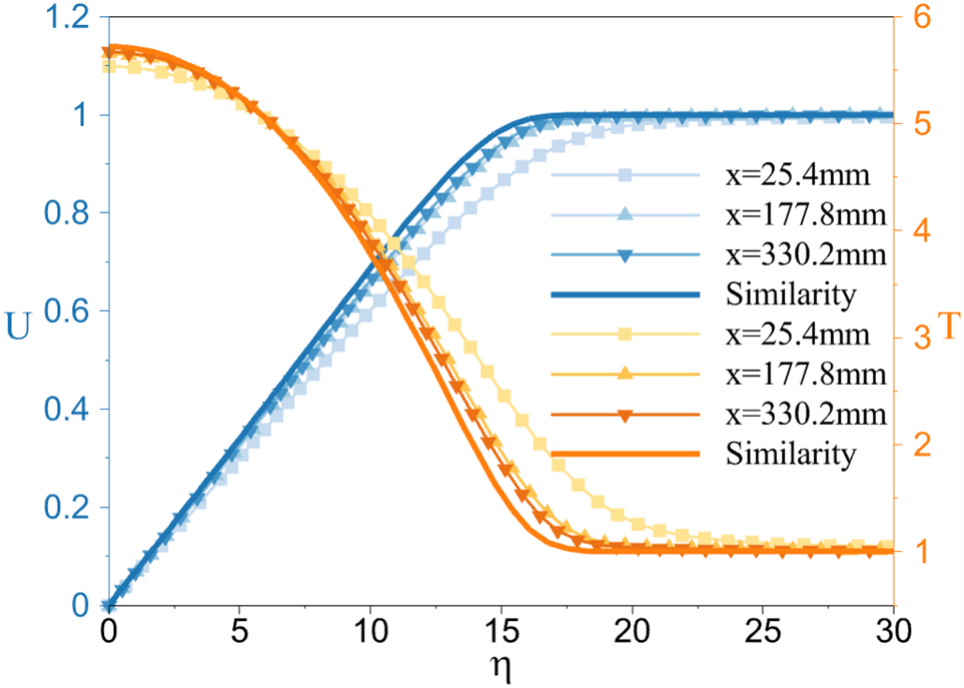
Validation of the normalized velocity and temperature distribution within the boundary layer with similarity solution obtained for the $M=6$ flow with the freestream temperature of 63.3 K. The normalization is done with the corresponding edge values.

Firstly, the mean flow is solved until the convergence is obtained with a grid with 3940 elements in the stream-wise direction and 425 elements in the wall-normal direction. The grid independence study for this geometry and flow condition is conducted in Ref.^28^. Hence, it is not repeated in the present work. Figure 4 illustrates the Mach contour in the computational domain. In this study, the nose radius is selected to be small enough to diminish the stabilization effect of the entropy layer^17,57,59,60^. Additionally, the steady flow boundary layer velocity and temperature profiles are compared with the similarity solution^61,62^ in Fig. 5, and an excellent agreement is found. The similarity parameter $\eta$ is defined as:

14 $$\begin{aligned} \eta =\frac{y_{n}}{\sqrt{\frac{\nu _{e}s_x}{U_{e}}}}, \end{aligned}$$

where $y_{n}$ is the wall-normal distance, $s_x$ is the distance along the surface of the wedge, and $\nu _{e}$ and $U_{e}$ are the kinematic viscosity and the velocity at the boundary layer edge. The velocity and temperature are nondimensionalized with the corresponding edge values.

To investigate the effect of the LC-LM boundary layer transition control method, we conducted mean flow simulations for four distinct cases: adiabatic wall condition case, LC strip case, LM coating case, and LC-LM treatment case. The names of the cases and locations of the LC strip and LM coating are given in Table 1.

**Table 1:** Location of the local cooling strip and metasurface coating.

Case	Cooling location (mm)	Metasurface location (mm)
AW	–	–
LC	25.4–152.4	–
LM	–	165.1–292.1
LC-LM	25.4–152.4	165.1–292.1

For the LC and LC-LM cases, the temperature distribution across the cooling strip is described as follows:

15 $$\begin{aligned} T_{w}= {\left\{ \begin{array}{ll}T_{ad}+\frac{T_{ad}\times 0.4}{2} \tanh \left( 0.3937\left( x-25.4\right) \right) +\frac{T_{ad}\times 0.4}{2}, &{} 25.4\le x \le 88.9,\\ T_{ad}-\frac{T_{ad}\times 0.4}{2} \tanh \left( 0.3937\left( x-152.4\right) \right) +\frac{T_{ad}\times 0.4}{2}, &{} 88.9<x\le 152.4.\end{array}\right. } \end{aligned}$$

**Figure 6:**
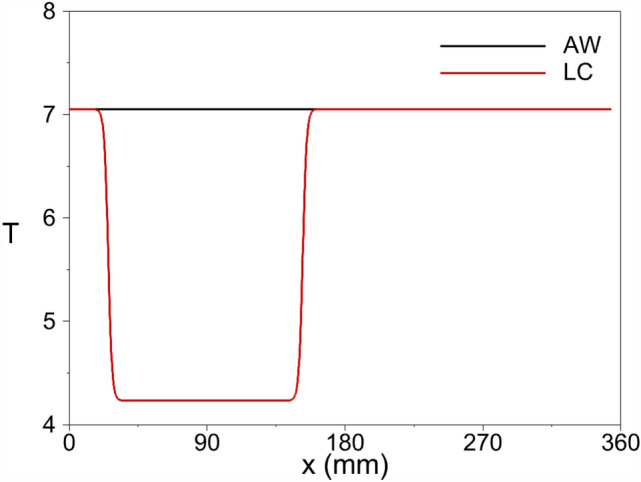
The wall temperature distribution for adiabatic wall (AW) and local cooling (LC) cases.

The resultant wall temperature profile for the cases with a cooling strip and the adiabatic wall case is illustrated in Fig. 6. The minimum wall temperature over the cooling strip corresponds to 60% of the adiabatic wall temperature. To avoid discontinuity in the temperature profile over the cooling strip, a tanh(*x*) function is used.

The pore radius, $b^{*}$, is 50 $\upmu$m for the LM and LC-LM cases. The distance between two consecutive pores, $s^{*}$, is taken as 100 $\upmu$m and the depth of the pore, $H^{*}$, is taken as 450 $\upmu$m. For the unsteady simulation, acoustic disturbances were introduced from the inlet boundary. To investigate the disturbances associated with the second mode, the frequency of the disturbances is chosen as 246 kHz, corresponding to the frequency band of the disturbances associated with the second mode^39^. The dimensionless frequency corresponding to 246 kHz is $F=0.63 \times 10^{-4}$, where the dimensionless frequency is defined as:

16 $$\begin{aligned} F=\frac{2\pi f \nu _{\infty } }{U_{\infty }^2}. \end{aligned}$$

Herein, *f* is the dimensional frequency, $\nu _{\infty }$ and $U_{\infty }$ are freestream kinematic viscosity and velocity, respectively. To ensure the instability wave amplitude growth remains within the linear regime, the amplitude of the forcing freestream acoustic waves was set to a small value of $2 \times 10^{-5}$, nondimensionalized by the freestream pressure.

**Figure 7:**
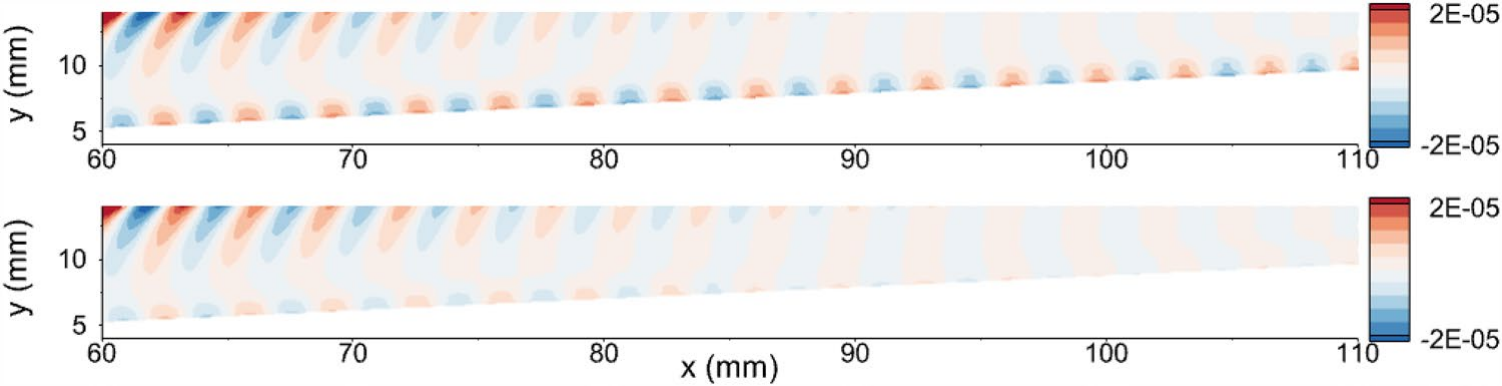
The pressure disturbance field in the region of 60 mm $<x<110$ mm. Top to bottom: adiabatic wall case, local cooling case.

Once the acoustic disturbances are introduced from the boundary, the unsteady simulations are run, and the results are obtained. The resultant flowfield variables are then subtracted from the steady flowfield variables, and the disturbance field is obtained. Figure 7 shows the pressure disturbance contours for AW and LC strip cases in the 60 mm $<x<110$ mm region. The region corresponding to the cooling strip is 25.4 mm $<x<152.4$ mm. In the AW case, the disturbance amplitude remains relatively constant. However, introducing a local cooling strip has a notable stabilizing effect on the disturbance. Consequently, the amplitude of the wave decreases noticeably over the cooling strip. For the LC case, the wall temperature drops along the cooling strip. This leads to a reduction in both the temperature within the boundary layer and the thickness of the boundary layer downstream of the LC strip. Correspondingly, the thickness of the region defined by the relative sonic line decreases, which may be the reason for inhibited second mode growth^24,63^.

**Figure 8:**
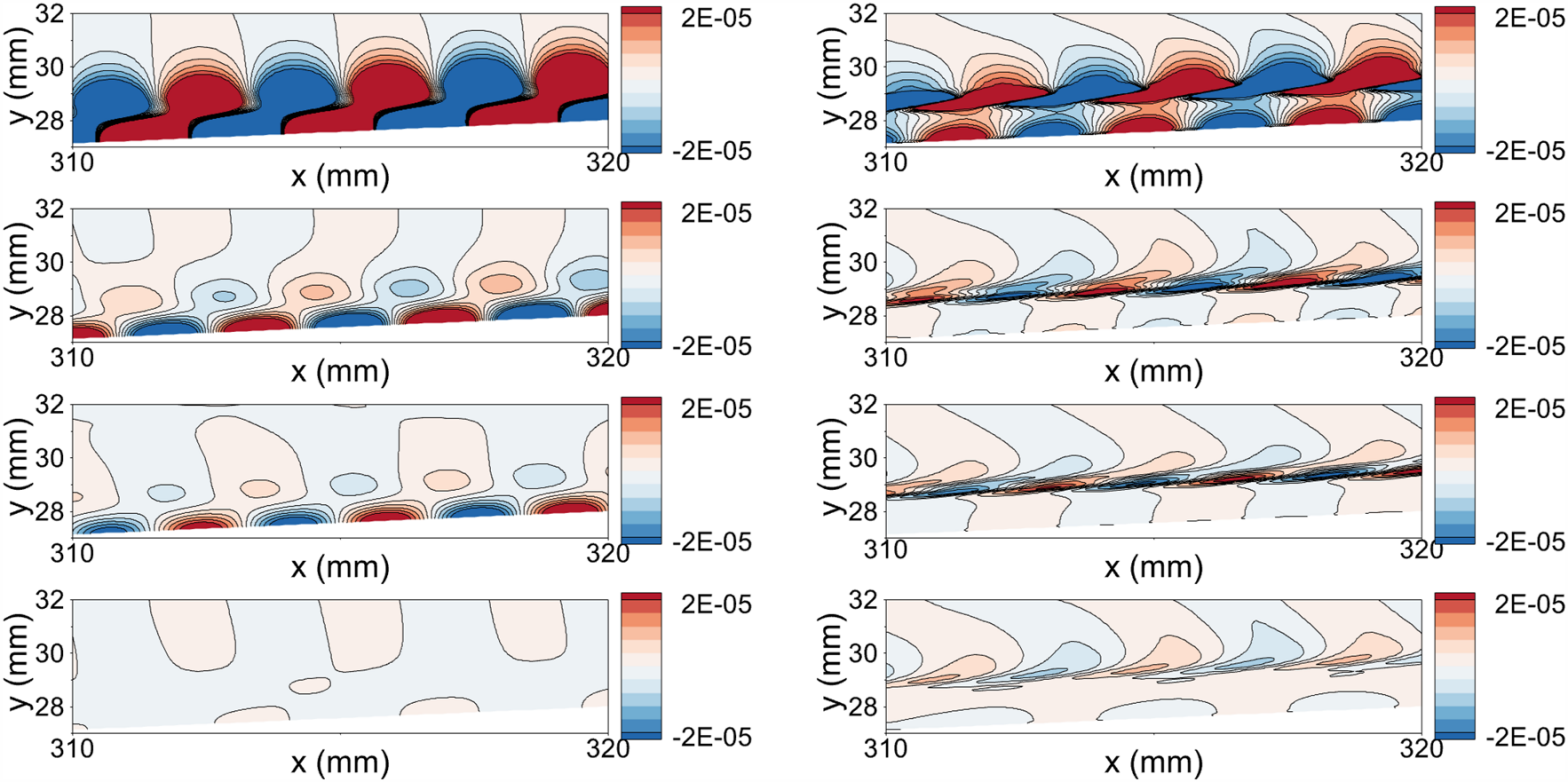
The pressure and density disturbance field in the region of 310 mm $<x<320$ mm. The left plots are pressure disturbance field and the right figures are density disturbance field. Top to bottom: adiabatic wall case, local cooling case, local metasurface case, and local cooling-local metasurface treatment case.

Figure 8 shows the pressure and density disturbance contours in 310 mm $<x<320$ mm, corresponding to the region after the LM coating. The adiabatic wall case is illustrated in the top row in Fig. 8. The pressure disturbance grows and forms the two-cell structure, a typical pattern for Mack second mode^64^. In the figure, the two-cell structure is already formed for the adiabatic wall case. When the density disturbance field is investigated, we observe the rope-like structures forming around the sonic line. As the disturbance evolves downstream, the density disturbances start to overlap to form the rope-like structures. These structures are observed for mode S. Hence, it can be observed in both first and second modes. For the LC case (second row from the top), the two-cell structure formation is delayed due to the stabilization over the cooling strip. The late two-cell formation indicates that the synchronization point moved downstream due to the cooling introduced by the local cooling strip. Since the first and second modes are directly related to the synchronization point, the second mode is observed at a location later than in the AW case. The density disturbance field for LC case supports the synchronization point observation. The density oscillation amplitudes show that the formation of the rope-like structures are delayed similar to two-cell structures. For the LM coating (third row from the top), the coating induces wall-normal velocity coupled with pressure disturbance. Each unit cell over the metasurface region traps the wave temporarily. This results in a suction/blowing effect over the metasurface unit cells due to the non-zero wall-normal velocity component. The resultant interactions inhibit the second mode development and significantly reduce the amplitude of the instantaneous wall pressure over the metasurface region. Thus, the amplitude of the disturbance on the wall at the end of the domain in the LM case is lower than that measured in the AW and LC cases. The density disturbance contours also indicate the stabilization of the second mode. As a result, the amplitude of the density disturbances decreased. For the LC-LM case (last row), the disturbance amplitude reaching the LM coating is significantly lower than in the LM case because of the stabilization over the LC strip. Over the LM coating, the disturbances are further stabilized and reach an amplitude lower than the initial disturbance amplitude. Moreover, in the LC-LM case, two-cell structures are not observed within the computational domain. However, we still observe the rope-like structures in the computational domain. This indicates that the disturbance is not completely stabilized but its amplitude is significantly reduced. As a result, the boundary layer transition is significantly delayed for the given conditions and frequency.

**Figure 9:**
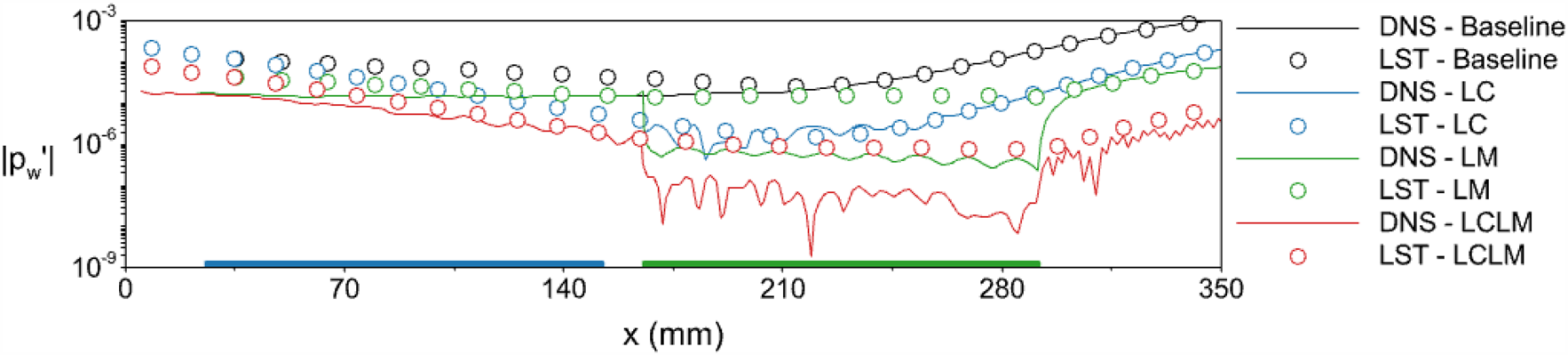
The wall pressure disturbance amplitude obtained from DNS and LST. The *y*-axis is in logarithmic scale. The blue line corresponds to the location of the LC strip, and the green line corresponds to the LM coating.

The instantaneous wall pressure disturbance amplitude obtained from DNS and LST is given in Fig. 9. The location of the local cooling strip is illustrated by a thin blue box over the *x*-axis, while the green box corresponds to the location of the local metasurface region. The plot is given in logarithmic scale to emphasize the order of the reduction in the wall pressure amplitude. The wall pressure amplitude, $A_p$, for LST is calculated as:

17 $$\begin{aligned} A_p=e^{\int _{x_0}^x\sigma (x,F)dx}, \end{aligned}$$

where $\sigma$ is the growth rate. For the AW case, the wall pressure of the disturbance field for the AW case is illustrated. The amplitude of the wall pressure starts to grow monotonically after $x>240$ mm. In the region of 290 mm $<x<340$ mm, the amplitude of the disturbance increased approximately 54 times compared to the initial amplitude introduced from the inlet for the AW case. LST results correlate well with the DNS results for the adiabatic wall case after $x>210$ mm. However, LST overpredicted the wall pressure upstream of this region. The discrepancy between LST and DNS is due to nonparallel flow near the nose region. Other researchers^24,54,65,66^ also observed a similar discrepancy.

For the LC strip case, the wall pressure amplitude initially decreases over the cooling strip due to the stabilized boundary layer. In the downstream of the LC strip ($x>152.4$ mm), the pressure disturbances recover and start to grow at approximately the same location as the adiabatic wall case ($x=210$ mm). However, the amplitude of the disturbance at that location is significantly lower (10 times) than the amplitude in the same location for the AW case. Downstream of this location, the amplitude of pressure disturbance increases monotonically. The maximum amplitude in the LC case is five times lower than the maximum amplitude observed in the AW case, which corresponds to approximately ten times higher than the initial disturbance.

For the LM coating case, the freestream parameters are identical to the adiabatic wall case up to the metasurface region. Thus, wall pressure oscillations are also identical. Over the LM region, the wall pressure is abruptly dropped by the metasurface unit cells. A similar trend is observed in several other studies^31,47,67^. After the initial juncture pressure drop, the disturbance is slightly weakened by the LM coating. However, after the end juncture, wall pressure recovers the values before the LM coating. After the wall pressure recovery, the monotonic increase starts in the wave amplitude. Since the metasurface inhibits the development of the second mode, the maximum instantaneous wall pressure amplitude in the LM case is approximately 14 times smaller than the AW case and only four times higher than the initial disturbance amplitude. The LST predictions for the LM case could not capture the abrupt pressure drop over the LM coating. Hence, the LST predictions are scaled to match the wall pressure at the initial juncture of the LM coating. Although LST overpredicted the amplitude, the trend of the disturbance growth aligns with the DNS results.

In the LC-LM case, the disturbance amplitude monotonically decreases over the cooling strip, similar to the LC case. Hence, the formation of the two-cell structures are delayed and the bottom half of the two cell structures exist in the flow field. When the disturbance reaches the metasurface region, the dominant part of the two-cell structures are still the bottom part as the upper part is not fully developed. The semitransparent surface over the metasurface region traps the bottom part of the wave. As a result the amplitude of the disturbance is further reduced. In the downstream of the end juncture of the LM coating, the disturbance amplitude is 200 times lower than the initial disturbance amplitude. Further downstream, the monotonic increase of the second mode starts; however, it reaches 20% of the initial disturbance amplitude at the end of the computational domain. We also checked if the disturbance ever exceeds the amplitude of the initial disturbance when the computational domain is longer for the LC-LM treatment case. The LST results from the extended domain indicated that the disturbance remains in the same order without substantial growth. These results illustrate that the combination of the LC strip with LM coating has significantly better stabilization than individual boundary layer transition control methods. In the case with the LC strip, the amplitude of the disturbance at the end of the computational domain, which corresponds to the maximum wall pressure amplitude in the flowfield, was 10 times smaller than the baseline case. The same ratio is 14 for the LM coating case. This intuitively leads to think that the combination of them should decrease the amplitude 140 times. However, the LC-LM method decreased the disturbance amplitude 240 times, which is significantly more than initial intuitive value.

**Figure 10:**
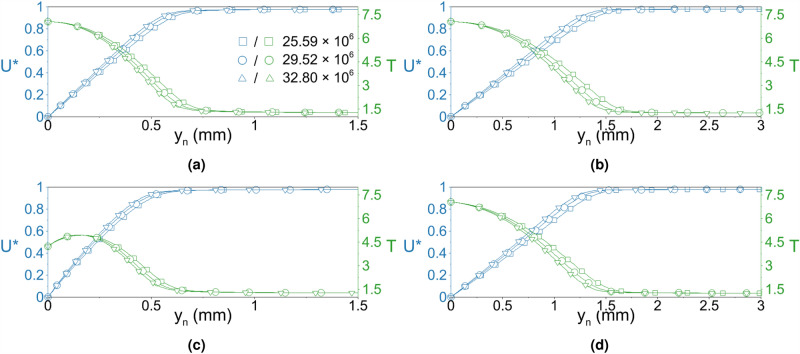
Boundary layer velocity and temperature profiles at (**a**) 50 mm (Baseline), (**b**) 300 mm (Baseline), (**c**) 50 mm (Local cooling), and (**d**) 300 mm (Local cooling).

In order to identify the effect of the Reynolds number on the stabilization, we conducted additional DNS simulations with varying Reynolds numbers. The Mach number is kept identical. However, the density of the air is changed to adjust the Reynolds number. The new unit Reynolds numbers are $29.52 \times 10^6/$m and $32.8 \times 10^6/$m. The steady simulations of the cases with new Reynolds numbers are obtained. The wall-normal velocity and temperature profiles of 3 different Reynolds numbers at $x=50$ mm and $x=300$ mm are given in Fig. 10 for AW and LC cases. The velocity and temperature profiles are scaled with the corresponding freestream values. The wall temperature is identical for all cases except the region over the local cooling strip. For the LC case, $x=50$ mm corresponds to the region over the local cooling strip. Thus, the boundary layer thickness is smaller than the AW case at $x=50$ mm. It is approximately 1 mm at $x=50$ mm for the AW case and 2 mm at the end of the domain where those boundary layer thicknesses are typical in the given conditions^39^. Due to the change in the freestream density, the boundary layer thickness decreased with increasing unit Reynolds number.

**Figure 11:**
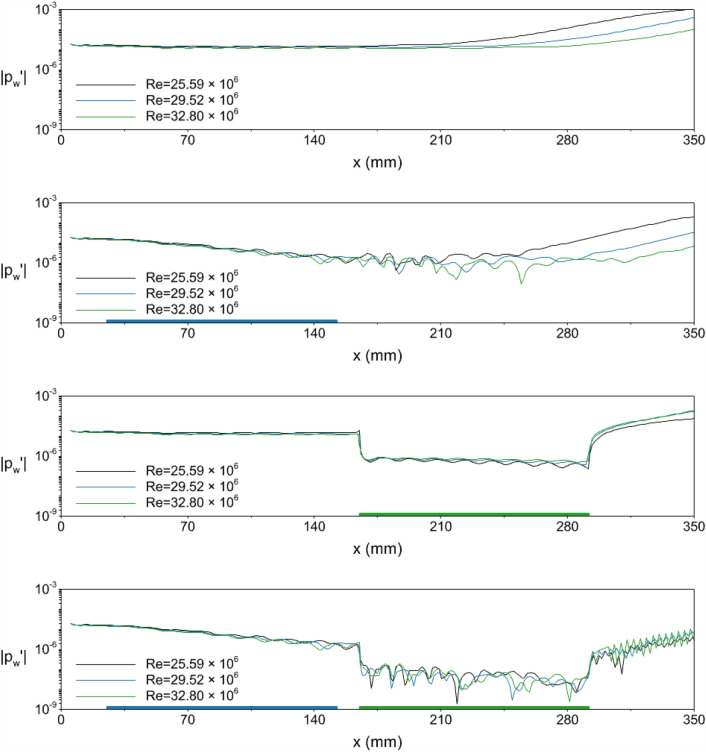
The amplitude of the wall pressure distribution obtained from DNS with varying Reynolds numbers. The *y*-axis is in logarithmic scale. Top to bottom: adiabatic wall (AW), local cooling (LC) strip, local metasurface (LM) coating, and local cooling-local metasurface (LC-LM) treatment. The blue line corresponds to the location of the LC strip, and the green line corresponds to the LM coating.

After the steady simulations, unsteady simulations are conducted. The dimensional frequency of the disturbance is kept identical in unsteady simulations. Thus, the dimensionless frequency is changed for each unit Reynolds number. The dimensionless frequencies are $F=0.546 \times 10^{-4}$ and $F=0.4914 \times 10^{-4}$ for unit Reynolds number of $29.52 \times 10^6/$m and $32.8 \times 10^6/$m, respectively. The resultant instantaneous wall pressure amplitudes with the new Reynolds numbers for all the cases are given in Fig. 11. The *y*-axis is in logarithmic scale. The thick blue line on the figure corresponds to the location of the LC strip, and the thick green line corresponds to the LM coating.

The AW case instantaneous wall pressure is given in the top row of Fig. 11. The amplitude of the disturbance at the end of the domain is significantly small with the increasing Reynolds number. The Reynolds number increase reduces the boundary layer thickness without affecting the freestream temperature or velocity. The resultant effect is similar to wall cooling, which decreases the boundary layer thickness and moves the synchronization point downstream. This, in turn, may delay the disturbance amplitude growth for a given frequency^17^. The amplification starts around at $x=168$ mm, $x=195$ mm, and $x=226$ mm for the $Re=25.59 \times 10^6/$m, $29.52 \times 10^6/$m and $32.8 \times 10^6/$m case, respectively.

In the second row of Fig. 11, the LC case is investigated. The location of the cooling strip is shown with a thin blue box over the *x*-axis. In all Reynolds numbers, the disturbance amplitude decreases over the cooling strip, and their decrement rates are identical. However, in a half cooling strip length away from the cooling region, the wall pressure amplitude increases monotonically for the $Re=25.59 \times 10^6/$m case. At the end of the domain, the highest Reynolds number has the minimum wall pressure amplitude for a given computational domain. The LM and LC-LM cases are investigated in the third and fourth row of Fig. 11. The location of the local metasurface is illustrated with a green box over the *x*-axis. Changing the Reynolds number did not affect the performance of the metasurface, and the instantaneous wall pressure fluctuation amplitudes were in the same order.

## Conclusion

Hypersonic boundary layer stability with local cooling strip, local metasurface region, and local cooling-local metasurface treatment are investigated over a 5-deg half-angle blunt wedge with 0.0254 mm nose radius. The freestream Mach number is taken as 6. We investigated the development of second mode disturbances in the hypersonic boundary layer using direct numerical simulations. The analysis of DNS results over the wedge revealed that local cooling and local metasurface individually stabilized the boundary layer, and disturbance growth was inhibited over the cooling strip or metasurface region. However, when the cooling strip is combined with the metasurface region, the stabilization characteristics significantly increase compared to the addition of the total stabilization over the individual local cooling case and local metasurface case. Additionally, the amplitude of the disturbances in the LC-LM case is reduced to a value lower than the initial amplitude of the disturbance. To investigate the stabilization characteristics of the LC-LM method with varying Reynolds numbers, the cases are run with three different Reynolds numbers. The simulations indicated that the performance of the LM coating remains identical with varying Reynolds numbers. These numerical simulations are the first simulations indicating the performance of the LC-LM boundary layer transition control method with varying Reynolds numbers, and they show that the LC-LM treatment may be a promising boundary layer control mechanism for stabilizing the hypersonic boundary layers.

## Data Availability

Data sets generated during the current study are available from the corresponding author upon reasonable request.
